# Supplementary figures and images for: Illegal use of natural resources in federal protected areas of the Brazilian Amazon
Source: PeerJ. 2017 Oct 10;5:e3902. doi: 10.7717/peerj.3902 (PMC5639874; doi:10.7717/peerj.3902)

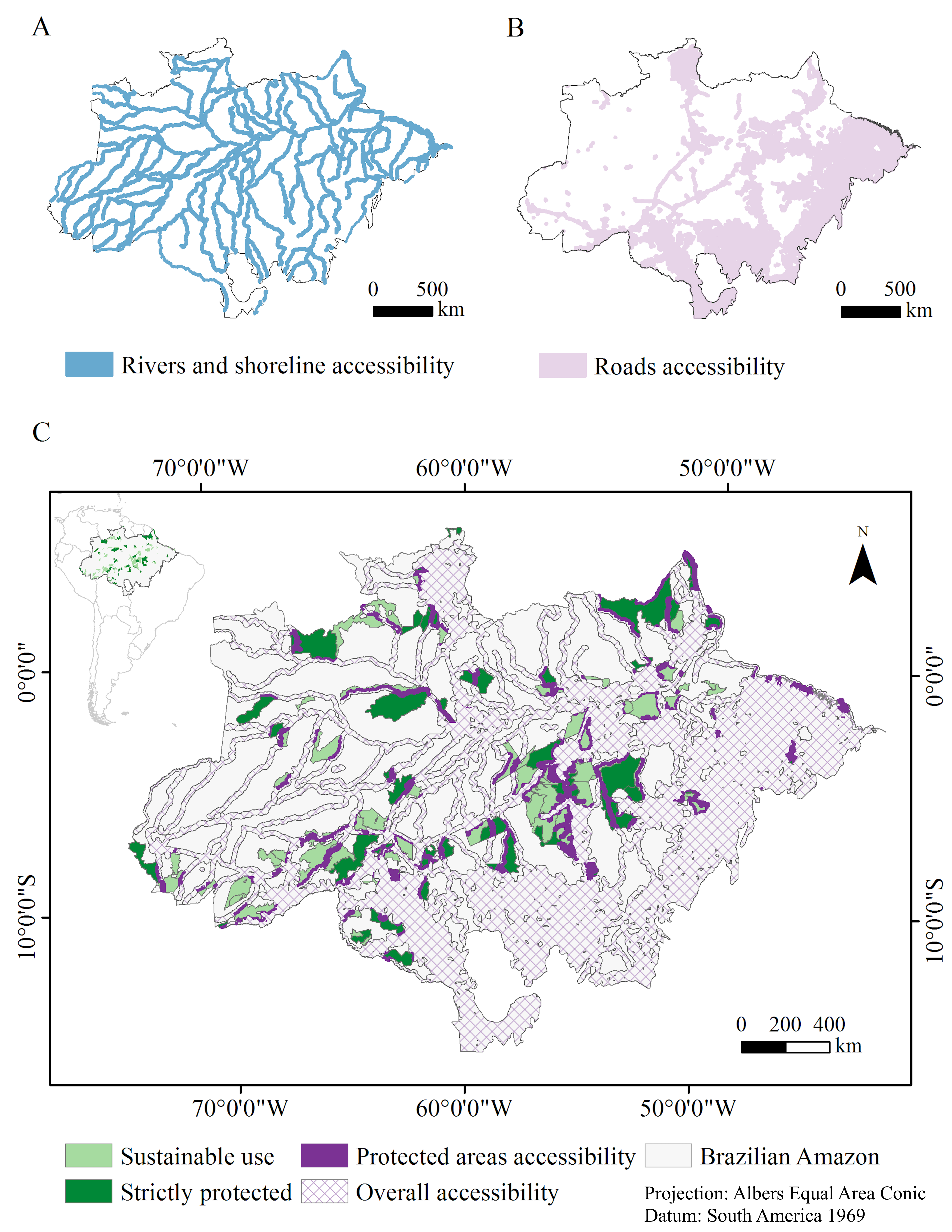

Supplement: Figure S1 — Accessibility of PAs defined as the intersection between the total area of a PA with the area of a 10 km buffer adjacent to roads and rivers located within or outside PAs. (A) rivers and shoreline accessibility; (B) roads accessibility; and (C) overall and PAs accessibility. [file peerj-05-3902-s001.png]

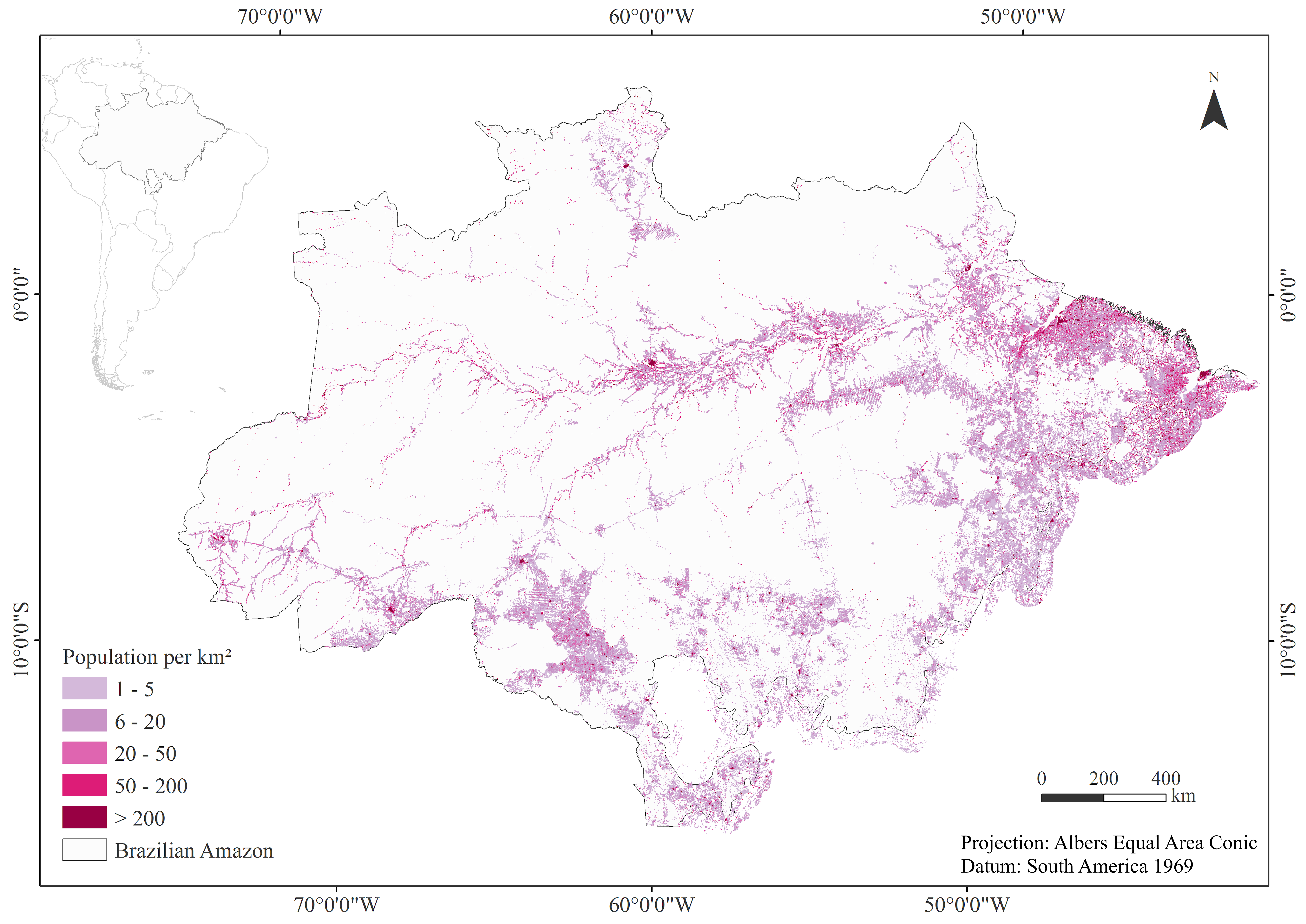

Supplement: Figure S2 — Population density map of the Amazon biome elaborated from the “Brazilian statistical grid” for visualization purposes. [file peerj-05-3902-s002.png]
