# Supplementary material for: Illegal use of natural resources in federal protected areas of the Brazilian Amazon
Source: PeerJ. 2017 Oct 10;5:e3902. doi: 10.7717/peerj.3902 (PMC5639874; doi:10.7717/peerj.3902)
Supplement: Table S1 — Amazon federal PAs evaluated (n = 118), Brazilian classification, IUCN classification, age in 2015, total area (km2), total number of illegal activities recorded (2010–2015), population density in a 50 km buffer from the perimeter of each PA and accessibility of a protected area (accessibility km2/total area km2). APA, Environmental Protected Area; ARIE, Area of Relevant Ecological Interest; ESEC, Ecological Reserve; PARNA, National Park; REBIO, Biological Reserve; FLONA, National Forest; RDS, Sustainable Development Reserve; RESEX, Extractive Reserve. [file peerj-05-3902-s003.docx]

| **Protected Area** | **Class** | **IUCN** | **Age (year)** | **Area (km²)** | **Illegal activities** | **Population density** | **Accessibility** |
| --- | --- | --- | --- | --- | --- | --- | --- |
| APA DO IGARAPE GELADO | Sustainable use | V | 26 | 232.85 | 0 | 12.911 | 1.000 |
| APA DO TAPAJOS | Sustainable use | V | 9 | 20,400.00 | 5 | 0.293 | 0.173 |
| ARIE JAVARI BURITI | Sustainable use | IV | 30 | 131.77 | 2 | 3.526 | 0.692 |
| ARIE PROJETO DINAMICA BIOLOGICA DE FRAGMENTOS FLORESTAIS | Sustainable use | IV | 30 | 31.80 | 0 | 3.303 | 1.000 |
| ARIE SERINGAL NOVA ESPERANCA | Sustainable use | IV | 16 | 25.74 | 4 | 8.344 | 1.000 |
| ESEC DA TERRA DO MEIO | Strictly protected | Ia | 10 | 33,730.00 | 43 | 0.160 | 0.211 |
| ESEC DE CARACARAI | Strictly protected | Ia | 33 | 867.95 | 13 | 1.268 | 0.729 |
| ESEC DE CUNIA | Strictly protected | Ia | 14 | 1,853.14 | 37 | 17.932 | 0.527 |
| ESEC DE JUTAI SOLIMOES | Strictly protected | Ia | 32 | 2,895.14 | 26 | 2.361 | 0.212 |
| ESEC DE MARACA | Strictly protected | Ia | 34 | 1,035.20 | 92 | 0.761 | 0.851 |
| ESEC DE MARACA JIPIOCA | Strictly protected | Ia | 34 | 602.53 | 10 | 1.412 | 0.999 |
| ESEC DO JARI | Strictly protected | Ia | 33 | 2,310.82 | 8 | 2.025 | 0.312 |
| ESEC JUAMI JAPURA | Strictly protected | Ia | 14 | 8,315.32 | 13 | 0.200 | 0.080 |
| ESEC NIQUIA | Strictly protected | Ia | 30 | 2,847.91 | 15 | 0.552 | 0.165 |
| ESEC RIO ACRE | Strictly protected | Ia | 34 | 790.94 | 0 | 0.297 | 0.375 |
| FLONA DE ALTAMIRA | Sustainable use | VI | 17 | 7,249.74 | 14 | 0.234 | 0.193 |
| FLONA DE ANAUA | Sustainable use | VI | 10 | 2,594.03 | 8 | 0.993 | 0.337 |
| FLONA DE BALATA TUFARI | Sustainable use | VI | 13 | 10,800.00 | 26 | 2.282 | 0.080 |
| FLONA DE CARAJAS | Sustainable use | VI | 17 | 3,912.63 | 40 | 10.222 | 0.666 |
| FLONA DE CAXIUANA | Sustainable use | VI | 54 | 3,179.51 | 13 | 3.124 | 0.321 |
| FLONA DE HUMAITA | Sustainable use | VI | 17 | 4,731.59 | 76 | 1.656 | 0.153 |
| FLONA DE ITAITUBA I | Sustainable use | VI | 17 | 2,128.92 | 6 | 0.298 | 0.332 |
| FLONA DE ITAITUBA II | Sustainable use | VI | 17 | 3,977.56 | 53 | 3.805 | 0.764 |
| FLONA DE JACUNDA | Sustainable use | VI | 11 | 2,212.20 | 55 | 1.239 | 0.119 |
| FLONA DE MULATA | Sustainable use | VI | 14 | 2,166.04 | 10 | 1.132 | 0.121 |
| FLONA DE PAU ROSA | Sustainable use | VI | 14 | 9,881.87 | 28 | 0.784 | 0.366 |
| FLONA DE RORAIMA | Sustainable use | VI | 26 | 1,696.29 | 31 | 0.731 | 0.588 |
| FLONA DE SANTA ROSA DO PURUS | Sustainable use | VI | 14 | 2,315.57 | 2 | 0.323 | 0.348 |
| FLONA DE SAO FRANCISCO | Sustainable use | VI | 14 | 211.48 | 0 | 0.184 | 0.000 |
| FLONA DE SARACA TAQUERA | Sustainable use | VI | 26 | 4,412.88 | 58 | 5.549 | 0.505 |
| FLONA DE TEFE | Sustainable use | VI | 26 | 8,651.27 | 58 | 2.523 | 0.155 |
| FLONA DO AMANA | Sustainable use | VI | 9 | 6,825.61 | 2 | 0.249 | 0.099 |
| FLONA DO AMAPA | Sustainable use | VI | 26 | 4,603.59 | 42 | 1.668 | 0.181 |
| FLONA DO AMAZONAS | Sustainable use | VI | 26 | 19,440.00 | 0 | 0.314 | 0.143 |
| FLONA DO BOM FUTURO | Sustainable use | VI | 27 | 973.85 | 46 | 21.751 | 0.136 |
| FLONA DO CREPORI | Sustainable use | VI | 9 | 7,403.96 | 10 | 0.451 | 0.018 |
| FLONA DO IQUIRI | Sustainable use | VI | 7 | 14,730.00 | 28 | 0.710 | 0.237 |
| FLONA DO ITACAIUNAS | Sustainable use | VI | 17 | 1,367.01 | 29 | 1.106 | 0.717 |
| FLONA DO JAMANXIM | Sustainable use | VI | 9 | 13,020.00 | 97 | 1.047 | 0.648 |
| FLONA DO JAMARI | Sustainable use | VI | 31 | 2,221.57 | 52 | 6.215 | 0.904 |
| FLONA DO JATUARANA | Sustainable use | VI | 13 | 5,694.28 | 2 | 0.605 | 0.189 |
| FLONA DO MACAUA | Sustainable use | VI | 27 | 1,763.47 | 0 | 0.185 | 0.000 |
| FLONA DO PURUS | Sustainable use | VI | 27 | 2,561.23 | 28 | 1.389 | 0.126 |
| FLONA DO TAPAJOS | Sustainable use | VI | 41 | 5,306.21 | 22 | 12.515 | 0.529 |
| FLONA DO TAPIRAPE AQUIRI | Sustainable use | VI | 26 | 1,965.06 | 5 | 1.868 | 0.473 |
| FLONA DO TRAIRAO | Sustainable use | VI | 9 | 2,575.29 | 59 | 1.956 | 0.794 |
| FLONA MAPIA INAUINI | Sustainable use | VI | 26 | 3,689.50 | 1 | 1.081 | 0.000 |
| PARNA DA AMAZONIA | Strictly protected | II | 41 | 10,660.00 | 71 | 3.277 | 0.257 |
| PARNA DA SERRA DO DIVISOR | Strictly protected | II | 26 | 8,375.60 | 199 | 2.551 | 0.092 |
| PARNA DA SERRA DO PARDO | Strictly protected | II | 10 | 4,454.13 | 19 | 0.871 | 0.137 |
| PARNA DE ANAVILHANAS | Strictly protected | II | 34 | 3,502.43 | 90 | 75.902 | 0.610 |
| PARNA DE PACAAS NOVOS | Strictly protected | II | 36 | 7,086.70 | 0 | 4.857 | 0.242 |
| PARNA DO CABO ORANGE | Strictly protected | II | 35 | 6,573.28 | 122 | 2.138 | 0.730 |
| PARNA DO JAMANXIM | Strictly protected | II | 9 | 8,598.07 | 62 | 0.427 | 0.505 |
| PARNA DO JAU | Strictly protected | II | 35 | 23,670.00 | 16 | 0.122 | 0.096 |
| PARNA DO JURUENA | Strictly protected | II | 9 | 19,580.00 | 70 | 0.220 | 0.329 |
| PARNA DO MONTE RORAIMA | Strictly protected | II | 26 | 1,167.49 | 11 | 1.035 | 0.000 |
| PARNA DO PICO DA NEBLINA | Strictly protected | II | 36 | 22,530.00 | 14 | 1.231 | 0.165 |
| PARNA DO RIO NOVO | Strictly protected | II | 9 | 5,381.57 | 0 | 0.164 | 0.199 |
| PARNA DO VIRUA | Strictly protected | II | 17 | 2,149.51 | 4 | 0.703 | 0.658 |
| PARNA DOS CAMPOS AMAZONICOS | Strictly protected | II | 9 | 9,613.27 | 68 | 0.795 | 0.427 |
| PARNA MAPINGUARI | Strictly protected | II | 7 | 17,770.00 | 187 | 8.764 | 0.107 |
| PARNA MONTANHAS DO TUMUCUMAQUE | Strictly protected | II | 13 | 38,650.00 | 15 | 0.500 | 0.225 |
| PARNA NASCENTES DO LAGO JARI | Strictly protected | II | 7 | 8,127.53 | 10 | 0.605 | 0.250 |
| PARNA SERRA DA CUTIA | Strictly protected | II | 14 | 2,835.03 | 0 | 0.496 | 0.000 |
| PARNA SERRA DA MOCIDADE | Strictly protected | II | 17 | 3,599.44 | 1 | 0.134 | 0.014 |
| REBIO DE UATUMA | Strictly protected | Ia | 25 | 9,387.32 | 40 | 0.337 | 0.188 |
| REBIO DO ABUFARI | Strictly protected | Ia | 33 | 2,238.67 | 316 | 0.735 | 0.640 |
| REBIO DO GUAPORE | Strictly protected | Ia | 33 | 6,157.76 | 74 | 2.757 | 0.154 |
| REBIO DO GURUPI | Strictly protected | Ia | 27 | 2,712.01 | 137 | 6.243 | 0.960 |
| REBIO DO JARU | Strictly protected | Ia | 36 | 3,468.64 | 158 | 2.648 | 0.328 |
| REBIO DO LAGO PIRATUBA | Strictly protected | Ia | 35 | 3,924.75 | 33 | 3.060 | 0.410 |
| REBIO DO RIO TROMBETAS | Strictly protected | Ia | 36 | 4,077.59 | 122 | 0.737 | 0.401 |
| REBIO DO TAPIRAPE | Strictly protected | Ia | 26 | 992.73 | 13 | 2.459 | 0.646 |
| REBIO NASCENTES DA SERRA DO CACHIMBO | Strictly protected | Ia | 10 | 3,421.96 | 70 | 0.586 | 0.914 |
| RDS DE ITATUPA BAQUIA | Sustainable use | VI | 10 | 644.42 | 3 | 3.046 | 0.690 |
| RESEX ARAPIXI | Sustainable use | VI | 9 | 1,337.12 | 8 | 1.831 | 0.672 |
| RESEX ARIOCA PRUANA | Sustainable use | VI | 10 | 838.17 | 11 | 13.402 | 0.806 |
| RESEX AUATI PARANA | Sustainable use | VI | 14 | 1,469.49 | 10 | 1.564 | 0.000 |
| RESEX BARREIRO DAS ANTAS | Sustainable use | VI | 14 | 1,061.99 | 1 | 0.266 | 0.000 |
| RESEX CHICO MENDES | Sustainable use | VI | 25 | 9,315.43 | 93 | 14.837 | 0.291 |
| RESEX CHOCOARE MATO GROSSO | Sustainable use | VI | 13 | 27.83 | 20 | 45.787 | 1.000 |
| RESEX DE CURURUPU | Sustainable use | VI | 11 | 1,860.57 | 16 | 20.772 | 0.999 |
| RESEX DE SAO JOAO DA PONTA | Sustainable use | VI | 13 | 34.09 | 48 | 57.756 | 1.000 |
| RESEX DO ALTO JURUA | Sustainable use | VI | 25 | 5,379.49 | 15 | 1.269 | 0.227 |
| RESEX DO ALTO TARAUACA | Sustainable use | VI | 15 | 1,509.24 | 23 | 0.738 | 0.569 |
| RESEX DO BAIXO JURUA | Sustainable use | VI | 14 | 1,780.39 | 44 | 0.482 | 0.332 |
| RESEX DO CAZUMBA IRACEMA | Sustainable use | VI | 13 | 7,549.87 | 37 | 1.510 | 0.048 |
| RESEX DO CIRIACO | Sustainable use | VI | 23 | 81.07 | 22 | 48.872 | 1.000 |
| RESEX DO LAGO DO CAPANA GRANDE | Sustainable use | VI | 11 | 3,043.13 | 14 | 1.398 | 0.271 |
| RESEX DO LAGO DO CUNIA | Sustainable use | VI | 16 | 506.04 | 10 | 28.448 | 0.745 |
| RESEX DO MEDIO JURUA | Sustainable use | VI | 18 | 2,869.55 | 12 | 1.144 | 0.441 |
| RESEX DO MEDIO PURUS | Sustainable use | VI | 7 | 6,042.36 | 36 | 1.188 | 0.651 |
| RESEX DO QUILOMBO FLEXAL | Sustainable use | VI | 23 | 93.38 | 17 | 19.486 | 1.000 |
| RESEX DO RIO CAJARI | Sustainable use | VI | 25 | 5,324.05 | 42 | 3.929 | 0.395 |
| RESEX DO RIO DO CAUTARIO | Sustainable use | VI | 14 | 751.25 | 2 | 1.171 | 0.009 |
| RESEX DO RIO JUTAI | Sustainable use | VI | 13 | 2,755.16 | 32 | 0.862 | 0.516 |
| RESEX DO RIO OURO PRETO | Sustainable use | VI | 25 | 2,046.33 | 29 | 3.321 | 0.476 |
| RESEX GURUPA MELGACO | Sustainable use | VI | 9 | 1,455.74 | 11 | 3.391 | 0.162 |
| RESEX IPAU ANILZINHO | Sustainable use | VI | 10 | 558.35 | 18 | 7.159 | 1.000 |
| RESEX ITUXI | Sustainable use | VI | 7 | 7,763.30 | 4 | 0.201 | 0.228 |
| RESEX MAE GRANDE DE CURUCA | Sustainable use | VI | 13 | 366.79 | 60 | 62.564 | 0.999 |
| RESEX MAPUA | Sustainable use | VI | 10 | 937.48 | 0 | 4.144 | 0.000 |
| RESEX MARACANA | Sustainable use | VI | 13 | 301.80 | 48 | 47.593 | 1.000 |
| RESEX MARINHA DE ARAI PEROBA | Sustainable use | VI | 10 | 625.78 | 3 | 36.398 | 1.000 |
| RESEX MARINHA DE CAETE TAPERACU | Sustainable use | VI | 10 | 424.90 | 42 | 47.456 | 1.000 |
| RESEX MARINHA DE GURUPI PIRIA | Sustainable use | VI | 10 | 727.90 | 9 | 31.715 | 1.000 |
| RESEX MARINHA DE SOURE | Sustainable use | VI | 14 | 295.79 | 129 | 19.456 | 1.000 |
| RESEX MARINHA DE TRACUATEUA | Sustainable use | VI | 10 | 278.65 | 7 | 52.282 | 1.000 |
| RESEX RENASCER | Sustainable use | VI | 6 | 2,096.67 | 20 | 3.023 | 0.120 |
| RESEX RIO IRIRI | Sustainable use | VI | 9 | 3,989.98 | 10 | 0.217 | 0.635 |
| RESEX RIO UNINI | Sustainable use | VI | 9 | 8,496.93 | 0 | 0.369 | 0.539 |
| RESEX RIO XINGU | Sustainable use | VI | 7 | 3,030.05 | 36 | 0.061 | 0.670 |
| RESEX RIOZINHO DA LIBERDADE | Sustainable use | VI | 10 | 3,249.06 | 48 | 1.565 | 0.063 |
| RESEX RIOZINHO DO ANFRISIO | Sustainable use | VI | 11 | 7,361.44 | 31 | 1.511 | 0.076 |
| RESEX TAPAJOS ARAPIUNS | Sustainable use | VI | 17 | 6,775.21 | 41 | 11.065 | 0.081 |
| RESEX TERRA GRANDE PRACUUBA | Sustainable use | VI | 9 | 1,948.70 | 12 | 9.604 | 0.000 |
| RESEX VERDE PARA SEMPRE | Sustainable use | VI | 11 | 12,890.00 | 78 | 2.627 | 0.185 |
