# Supplementary material for: Illegal use of natural resources in federal protected areas of the Brazilian Amazon
Source: PeerJ. 2017 Oct 10;5:e3902. doi: 10.7717/peerj.3902 (PMC5639874; doi:10.7717/peerj.3902)
Supplement: Table S2 — Categorization of illegal activities considering the infraction framework, the number of occurrences of each type of infraction (according to Brazilian Federal Decree 6514 (2008)), and the main characteristics of illegal activities. [file peerj-05-3902-s004.docx]

| **Illegal activities categories** | **Types of illegal use of natural resources** | **Illegal activities (n)** | **Fines (US$)^a^** |
| --- | --- | --- | --- |
| Suppression or degradation of vegetation | To market, carry, or use chainsaw without authorization | 106 | 73,107.01 |
|  | Illegal trade in timber | 372 | 4,050,076.70 |
|  | Prevent natural regeneration of forests and other types of natural vegetation | 122 | 40,732,903.09 |
|  | Production of coal without authorization or in disagreement with that obtained | 5 | 2,209.60 |
|  | Suppression or degradation of vegetation (e.g. deforestation, selective logging, logging of endangered species) | 937 | 142,600,414.46 |
|  | Make use of fire without authorization or in disagreement with that obtained (e.g. use of fire that could cause forest fires) | 43 | 879,103.54 |
| Subtotal | | 1,585 | 188,337,814.39 |
| Illegal fishing | Fishing in strictly protected areas or in prohibited locations, outside the allowed period and above established quantities or sizes in sustainable-use areas | 1,160 | 3,966,355.11 |
| Subtotal | | 1,160 | 3,966,355.11 |
| Hunting activities | Hunting in strictly protected areas or for commercial purposes in sustainable-use areas | 769 | 17,688,522.10 |
|  | Abuse or mistreatment of animals (e.g. transport of animals in unhealthy conditions) | 1 | 631.31 |
| Subtotal | | 770 | 17,689,153.41 |
| Illegal mining | Mining in strictly protected areas and extractive reserves (e.g. sustainable-use areas where the activity is not allowed), and without authorization or in disagreement with the authorization | 202 | 1,107,001.26 |
| Subtotal | | 202 | 1,107,001.26 |
| Irregular occupation or construction | Irregularly occupying areas of a protected area for housing or enterprises. Build buildings where it is not allowed, without authorization or in disagreement with the authorization (e.g. to build a hotel in a national park without authorization) | 175 | 7,809,092.17 |
| Subtotal | | 175 | 7,809,092.17 |
| Practices and conduct in disagreement with regulations or category of AP | Causes damage to protected area (in case it can not be framed in any other type) | 2 | 631.31 |
|  | Realize conduct in disagreement with any specific regulations (e.g., exceed the established limits for visitation) | 10 | 5,145.20 |
|  | Entering motorized vehicles in areas not allowed | 42 | 18,686.87 |
|  | Enter the protected area without authorization | 82 | 52,714.65 |
| Subtotal | | 136 | 77,178.03 |
| Against environmental administration | Presentation of false information | 8 | 648,810.92 |
|  | Embargo noncompliance | 56 | 3,088,266.73 |
|  | Notification noncompliance | 33 | 617,859.22 |
|  | Disrupt or impede enforcement | 32 | 137,310.61 |
|  | Conduct research of any nature without authorization | 2 | 7,260.10 |
|  | Unauthorized commercial use of image | 1 | 2,367.42 |
| Subtotal | | 132 | 4,501,875.00 |
| Illegal use of NTFPs and other resources | Collection of non-timber forest products in strictly protected areas or in disagreement with regulations in sustainable-use areas | 40 | 141,414.14 |
| Subtotal | | 40 | 141,414.14 |
| Agricultural and farming activities | Breeding of animals and agricultural crops in strictly protected areas or in disagreement with regulations in sustainable-use areas | 17 | 80,018.94 |
|  | Introduction of species for commercial purposes that have great potential for impact or biological invasion (e.g. buffalos, exotic fish) | 9 | 47,348.48 |
| Subtotal | | 26 | 127,367.42 |
| Pollution | To construct, renovate, expand, install or operate facilities, activities, works or services users of environmental resources, considered as effective or potentially polluting, without the license or authorization of the competent environmental agencies, in disagreement with the license obtained or contrary to legal norms and regulations | 9 | 848,642.68 |
|  | Pollution at inadequate levels (e.g. to cause pollution of any nature at levels that result or may result in damage to human health, or that lead to the death of animals or the significant destruction of biodiversity) | 2 | 6,628.79 |
|  | Production, use and storage of toxic or hazardous substances | 6 | 33,617.42 |
| Subtotal | | 17 | 888,888.89 |
| **Total** | | **4,243** | **224,646,139.84** |

^a^ All fines were imposed in Brazilian real (R$) and converted to American dollar (US$) by using an exchange rate of R$ 3.168: US $1 for the purpose of comparison with other studies. Dollar quotation on 03/31/2017.
