# Supplementary material for: Illegal use of natural resources in federal protected areas of the Brazilian Amazon
Source: PeerJ. 2017 Oct 10;5:e3902. doi: 10.7717/peerj.3902 (PMC5639874; doi:10.7717/peerj.3902)
Supplement: Table S3 [file peerj-05-3902-s005.docx]

| Variables | Classes ^a^ | Age ^b^ | Accessibility ^c^ | Population density ^d^ |
| --- | --- | --- | --- | --- |
| Classes | - | -0.32*** | 0.10 | 0.24** |
| Age | -0.32*** | - | 0.02 | 0.15 |
| Accessibility | 0.10 | 0.02 | - | 0.46*** |
| Population density | 0.24** | 0.15 | 0.46*** | - |

Notes: ^a^ Class of protected areas (Sustainable use and Strictly protected); ^b^ Age of protected area creation (creation until 2015) log transformed (log_10_); ^c^ Accessibility of protected area square root transformed; ^d^ Population density in a 50 km buffer from the perimeter of each PA log transformed (log_10_ × 10^5^). Significance values: **p < 0.01, ***p < 0.001.
