# Supplementary material for: Illegal use of natural resources in federal protected areas of the Brazilian Amazon
Source: PeerJ. 2017 Oct 10;5:e3902. doi: 10.7717/peerj.3902 (PMC5639874; doi:10.7717/peerj.3902)
Supplement: Table S4 — Comparison between the number of illegal activities in the Brazilian Amazon federal PAs, and the PA location (coastal/marine, n = 13; or terrestrial, n = 105). The test was run separately for all illegal activities, hunting activities, illegal fishing, and flora degradation. [file peerj-05-3902-s006.docx]

| **PA’s components** | **Terrestrial PAs** | **Coastal/marine PAs** | **W^a^** |
| --- | --- | --- | --- |
| PAs number | 105 | 13 |  |
| PAs area (km²) | 584899.07 | 16044.65 |  |
| All Illegal activities | 3696 | 547 | 232^*^ |
| Hunting activities | 745 | 25 | 554^+^ |
| Illegal fishing | 788 | 1551 | 56^*^ |
| Flora degradation | 1551 | 34 | 649^+^ |

Notes: ^a^ Mann-Whitney U test performed with the total number of illegal activities (all illegal activities, hunting activities, illegal fishing, and flora degradation) divided by the number of years (n = 6) and the area of the PA (km²), and log_10_ ((illegal activities × 10^5^) + 1) transformed; ^+^ Not significant; ^*^ p < 0.001.
